# Aneurysmal bone cyst primary - about eight pediatric cases: radiological aspects and review of the literature

**DOI:** 10.11604/pamj.2013.15.111.2117

**Published:** 2013-07-28

**Authors:** Meryem Boubbou, Karima Atarraf, Lamiae Chater, Abderrahmane Afifi, Siham Tizniti

**Affiliations:** 1 Department of Radiology, CHU Hassan II, Fez, Morrocco; 2 Department of Pediatric Surgey, CHU Hassan II, Fez, Morrocco

**Keywords:** Bone cyst, aneurysm, osteolytic lesion

## Abstract

The aneurysmal bone cyst is a pseudotumoral lesion that can take several aspects. This is a rare lesion representing 1% of bone tumors. It appears usually during the first 30 years of life. The pathogenesis is that of a process of “dysplasia/hyperplasia”, favored by a circulatory deficiency and hemorrhage within the lesion and the phenomena of osteoclasis. The objective of this work is to illustrate with analysis, the specific forms and atypical aneurysmal bone cyst which often pose a diagnostic challenge requiring radiological investigation with histological confirmation. We report eight pediatric cases of aneurysmal cysts collected over a period of 3 years, 3 boys and 5 girls. All patients had standard radiographs. MRI was performed in three patients. The diagnosis was confirmed histologically. The atypia has been in the seat: fibula (1 case), metaphyseal (2 cases), diaphyseal (4 cases) and metatarsal (1 case). Aneurysmal bone cyst is a rare benign tumor with predilection to the metaphysis of long bones. Atypical forms even fewer are dominated by the atypical seat.

## Introduction

Aneurysmal bone cysts (ABC) are nonneoplastic expansile lesions that may exist as a primary bone cyst or as a secondary lesion arising from other osseous conditions such as giant cell tumors or unicameral bone cysts [1]. The peak age of occurrence is in the second decade of life; approximately 80% occur within the first two decades.

The long bones (especially the tibia and femur) and vertebrae are the most common sites. However, aneurysmal bone cysts may occur in any bone. The male to female ratio is 1 to 1.3. Pain is the most common clinical symptom at presentation. Local swelling may develop as the lesion increases in size. Occasionally, the patient may present with a pathologic fracture within the aneurismal bone cyst where the cortex is compromised. Major radiographic features include dilated or aneurysmal cystic expansion of the involved bone with no significant matrix mineralization. The lesion tends to affect the metaphyses of long bones and the dorsal elements of the vertebrae. Sclerotic rims with periosteal new bone formation are common. The radiographic differential diagnosis includes unicameral bone cysts, giant cell tumors, osteosarcoma, and osteoblastoma (in vertebral lesions). The diagnosis must be based on histopathologic evidence [2]. Local recurrence rate after classic surgical procedures (curettage and grafting) is about 11.8%-30.8% [3]. The purpose of this article is to show some unusual atypical head of pathology and a review of the literature.

## Methods

**Patients:** From 2008 to 2011, eight patients were treated for ABCs of long and flat bones at Hassan II Hospital by means chirurgical. The following data were collected retrospectively by one author (K.A): patient age, sex, symptoms, osseous location, and type of treatment, treatment outcome, pathologic findings, and complications.

**ABC Typing and Staging:** All patients had undergone initial conventional radiography. Three of them had an MRI. Surgical biopsy with histologic examination was performed for all patients for confirmed diagnostic.

**Figure 1:**
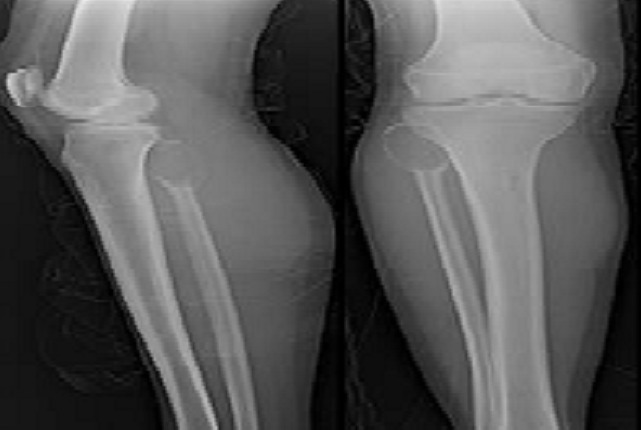
Radiograph showing an osteolytic lesion of the head of the fibula associated with intralesional few thin walls

**Figure 2:**
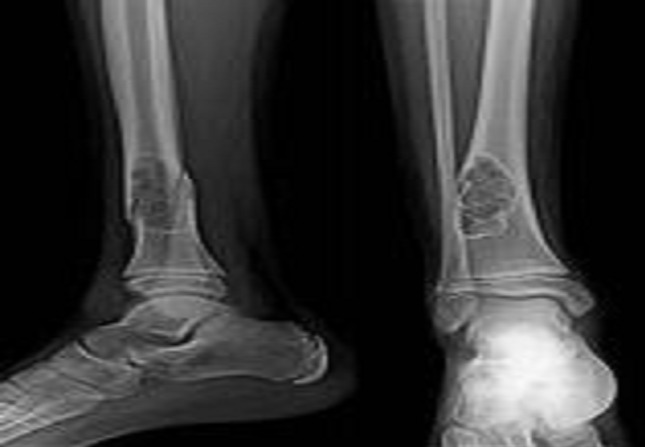
Radiograph showing an osteolytic lesion well demarcated from the lower end of the tibia containing the partitions associated with a spiral fracture

**Figure 3:**
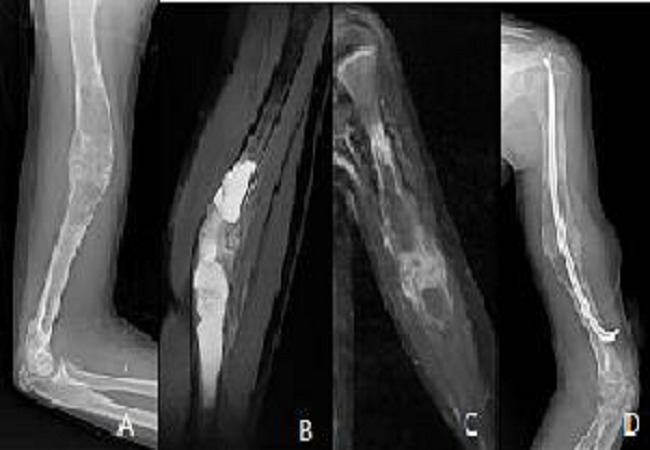
Radiograph shows osteolytic lesion very limited of the upper end of tibia

**Figure 4:**
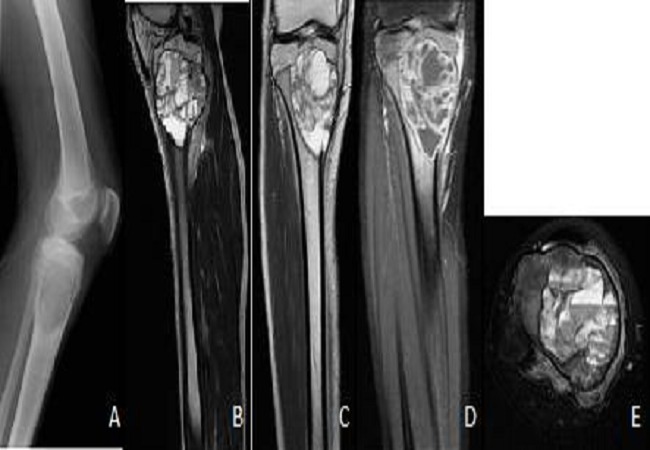
Radiography (A) showing an osteolytic lesion oval metaphyseal tibial very limited. Cuts MRI sagittal T1-weighted (B), coronal T2 (C) and T1 post contrast (D) and axial T2 FATSAT (E) showing the partitions after intralesional contrast enhanced liquid level typical of an aneurysmal cyst

**Figure 5:**
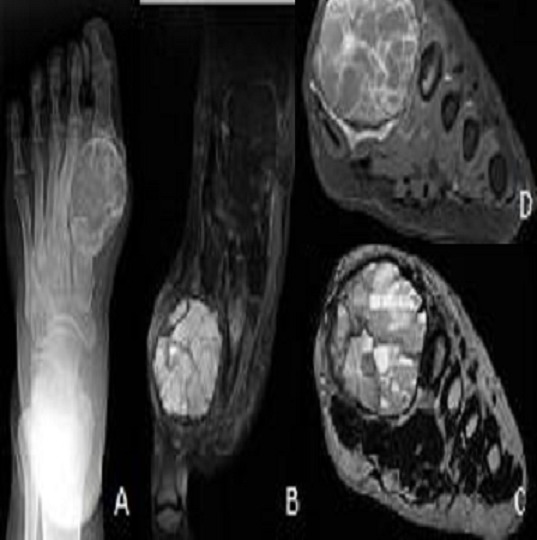
Radiography (A) showing a multiloculated osteolytic lesion of the metatarsal very limited. Cuts sagittal T1-weighted MRI (B), axial T2 (C) and FAT SAT T1 after contrast (D) showing an enhancement of intralesional walls with liquid level

**Figure 6:**
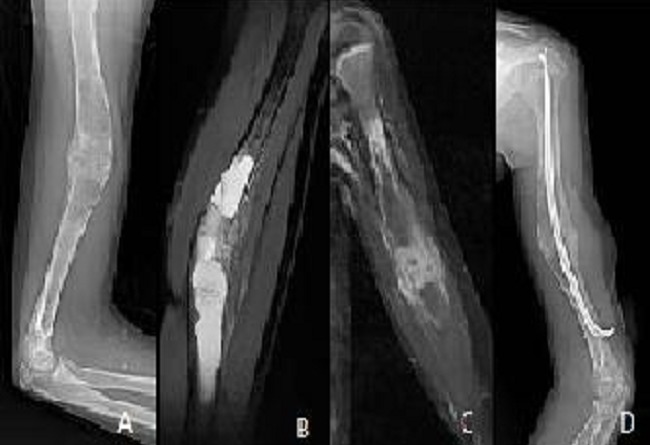
Radiography (A) showing a lesion: osteolytic multiloculated diaphyseal humeral fracture very limited. Cuts MRI T2 sagittal (B) Coronal T1 Fat Sat after contrast (C) showing a cystic lesion fractured in the center with peripheral enhancement. The child received surgical treatment (D)

**Imaging:** In radiography, the appearance was typical with the presence of a lesion lytic fan, responsible for a thinning of the cortex compared associated with thin walls intra-lesional (Figure 1, Figure 2, Figure 3). In MRI, the lesion is of heterogeneous signal containing a liquid level, with partitions sometimes taking the contrast (Figure 4, Figure 5, Figure 6).

**Treatment procedure:** Treatment consists mainly of surgical treatment which currently represents the treatment of choice. In most large series, the percentage of local recurrence after curettage Conventional is approximately 20%. In our series, all patients were operated without recurrence described so far.

## Results

**Figure 7:**
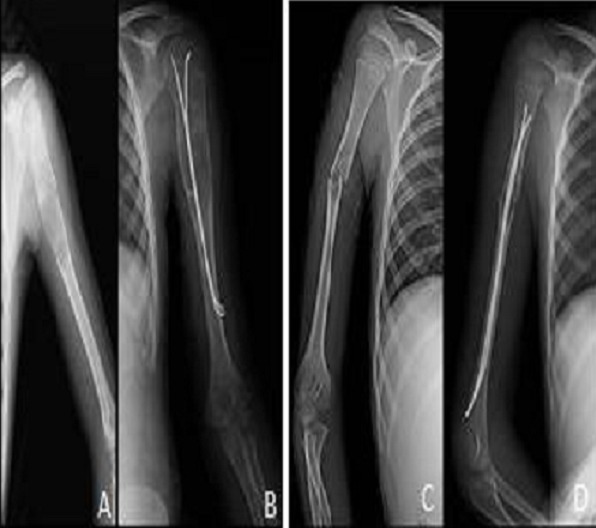
Radiography (A) showing a lesion: osteolytic multiloculated diaphyseal humeral very limited. The child was given a plug-curettage (B). Images C and D showing a lesion: osteolytic multiloculated diaphyseal humeral fracture very limited. The child received a pinning (D)

Eight patients (3 boys and 5 girls) were treated. Mean age at presentation was 10.3 years (range, 3-15 years). Symptoms at presentation included pain (8 patients) and pathologic fracture (four patients). Osseous locations were the proximal femur in one patient, proximal tibia in one, distal tibia in one, distal fibula in one, diaphyseal humerus in three, and metatarsal in one. All our patients were operated on. The surgical procedure consisted of curettage with bone replacement and plugging. The postoperative course was uneventful with almost complete radiographic healing (Figure 7).

## Discussion

ABCs represent approximately 1% -2% of all primary bone lesions that are sampled for biopsy [4], with a slight female preponderance, a male-to-female ratio of 1 - 1.04 and a median patient age of 13 years in large studies [5, 6]. In the present study, the median age was 9.6 years at presentation.

ABCs are most commonly found in the metaphysis of long tubular bones [5, 7–9]. Many theories have been postulated as to the etiology of these lesions, such as dynamic vascular changes within a newly formed part of the immature skeleton, with the possibility of some cases arising from preexisting bone lesions. This vascular change causes increased venous pressure, dilated vascular beds or thrombosis, or an arteriovenous fistula. The engorged vascular bed can lead to rapid resorption of spongy bone and erosion of cortical bone [6, 8, 9].

Essadki et al [10] hypothesize that ABCs are caused by the opposed direction of periosteal and medullary blood circulation. ABCs pass through different stages of development as part of their natural progression. The first stage consists of an early osteolytic lesion. The lesion then progresses into a mature characteristic cyst that eventually evolves into a late or calcified stage. Progression of ABCs is variable. They may have aggressive growth or grow slowly. They eventually mature and rarely undergo spontaneous regression [6, 11, 12].

CT can be helpful in the differentiation of ABCs from unicameral bone cysts when showing fluid-fluid levels within the cystic cavity, a finding nonspecific to but suggestive of ABC. Magnetic resonance imaging findings may also be highly suggestive of an ABC when a segmented, expansile, multiseptated lesion with fluid-fluid levels is demonstrated. Bone scintigraphy with technetium 99m typically shows a photon-deficient area with a rim of increased uptake [6, 8].

The high recurrence rate of ABCs indicates the need for new therapeutic modalities. Surgical treatment consists of excision of the lesion by means of curettage-with or without packing of bone chips-or en bloc resection. About 70% of ABCs show spontaneous ossification after intracapsular curettage. Marginal extracapsular excision is the treatment of choice, especially for recurrent lesions. Treatment of secondary lesions is directed against the underlying primary lesion [13]. Vergel De Dios et al [14] reported a 20% recurrence rate after curettage with or without bone grafting, usually within the first 2 postoperative years.

A local recurrence rate of 20% after curettage alone was reported by Campanacci et al [15]. The recurrence rate is increased in patients with a mitotic index greater than 7, patients who undergo curettage treatment alone (although repeat curettage usually provides a lasting cure), younger patients [8].

## Conclusion

Always think of an aneurysmal bone cyst to a tumor metaphyseal front fan 20. Should always seek a traumatic background and never forget to seek an underlying lesion (secondary ABC).
